# Neonatal Screening and the Clinical Outcome in Children with Sickle Cell Disease in Central India

**DOI:** 10.1371/journal.pone.0147081

**Published:** 2016-01-19

**Authors:** Dipti S. Upadhye, Dipty L. Jain, Yogesh L. Trivedi, Anita H. Nadkarni, Kanjaksha Ghosh, Roshan B. Colah

**Affiliations:** 1 National Institute of Immunohematology, (Indian Council of Medical Research), 13th floor, New Multistoried Building, K.E.M Hospital Campus, Parel, Mumbai, 400012, India; 2 Government Medical College, Nagpur, 440003, India; University of Naples Federico II, ITALY

## Abstract

**Background:**

Sickle cell disease (SCD) is a major health burden in India. The objective of the study was to establish a neonatal screening program and to understand the clinical course of children with SCD in central India.

**Methods and Findings:**

Pregnant mothers were screened for sickle hemoglobin using the solubility test. Babies were screened by high performance liquid chromatography if the mother was positive for sickle hemoglobin. The diagnosis was confirmed by molecular analysis. They received early prophylactic treatment and vaccination. Of 2134 newborns screened, 104 were sickle homozygous (SS), seven had sickle β-thalassemia (S-β thal) and 978 were sickle heterozygous (AS). The other hemoglobin abnormalities detected included HbS -δβ thalassemia-1, HbSD disease-2, HbE traits-5, β-thalassemia traits-4, alpha chain variants-3 and HbH disease-1.These babies were followed up regularly for hematological and clinical evaluation. Pain, severe anemia requiring blood transfusions and acute febrile illness were the major complications with 59.7, 45.1 and 42.6 cases per 100 person years. Fetal hemoglobin (HbF) levels were inversely associated with vaso-oclussive crisis (VOC) and severe anemia while presence of alpha thalassemia increased the rate of painful events and sepsis. Six early deaths occurred among the SS babies.

**Conclusion:**

A systematic follow up of this first newborn SCD cohort in central India showed that 47% of babies presented within 1 year of age. In spite of the presence of the Arab-Indian haplotype many babies had severe manifestations.

## Introduction

Studies on the natural history of sickle cell disease (SCD) from Jamaica and the USA have confirmed that the greatest morbidity and mortality occurs between 6 and 12 months and that early identification of affected infants by neonatal screening, careful follow up coupled with relatively simple measures decreased the mortality rate [1–3]. The sickle gene is prevalent in the tribal populations of India who are considered the original inhabitants living mainly in rural areas and in some non-tribal population groups like the scheduled castes and other backward classes belonging to a low socio economic status. Carrier frequencies range from 1–40% with the highest prevalence in central India [4].Our earlier report on newborn screening for sickle cell disease in central India showed a very high birth rate of sickle cell anemia babies (1.1%) with the highest incidence in the Mahar community (2.0%) [5]. Earlier studies from western India had shown that the disease was more severe in the non-tribal populations in Maharashtra than in the tribal groups of Gujarat [6]. More recent retrospective analysis of SCD in children from central India has shown that in some cases the disease can be as severe as in the African cohorts [7].

This is one of the first efforts to raise a cohort of SCD babies by newborn screening and follow them regularly to record the early clinical and hematological presentation in central India.

## Materials and Methods

### Ethical statement

The study was approved by the Institutional Ethics Committee Review Board- “Institutional Committee for Research on Human Subjects, National Institute of Immunohaematology (ICMR) (NIIH/IEC/21-2007)”, written informed consent was obtained from all participants and all investigations were conducted according to the principles expressed in the Declaration of Helsinki.

### Methods

Pregnant women were screened for sickle hemoglobin (HbS) using the solubility test at Govt. Medical College, Nagpur after a written informed consent was taken from them. Babies of all the mothers who had a positive solubility test were screened by high performance liquid chromatography (HPLC). Heel prick samples were collected in EDTA after birth or within 7 days of birth after a written informed consent from the parents and all the investigations on the babies and the parents samples were conducted according to the principles expressed in the Declaration of Helsinki. A complete blood count was done on an automated cell counter (Sysmex K-1000, Sysmex Corporation, Kobe, Japan). Hemoglobin (Hb) analysis was done using automated HPLC on the VARIANT™ Hemoglobin Testing System (Bio-Rad Laboratories, Hercules, CA, USA) using the sickle cell short programme and the β thal short programme during follow up. Molecular analysis was done to confirm the sickle and other genotypes [8]. Alpha genotyping was done using multiplex PCR (3.7 & 4.2) [9].

The gestational age at delivery, demographic details and neonatal complications were recorded. The babies with SCD were enrolled at the sickle cell clinic in Nagpur. Vaccination included conjugate vaccine for Haemophilus influenzae type B and 7-valent conjugate pneumococcal vaccine (CPV, Prevnar) within 4 weeks of birth. All the babies received oral penicillin V starting at 3 months of age and 23-valent polysaccharide pneumococcal vaccine (Pneumovax) was given after 2 years of age. Clinical crisis were defined according to previous published criteria [10–13]. A follow up visit card was given to the parents to record the dates of the clinical visit and the clinical events during each follow up. The parents were educated to recognize specific complications.

### Statistical analysis

Incidence rates are presented as the number of cases per 100 person years. The estimates were based on clinical events and follow up time. Descriptive statistics are presented as percentages and mean ± standard deviation. The p values are calculated by Fischer’s exact test (two tailed). A p value of 0.05 or less was considered significant.

## Results

### Patients

From 2009 to 2012, 10,181 pregnant women were screened and 2134 newborn babies were tested of whom 1040 (48.7%) were normal, 978 (45.8%) were sickle cell traits, 104 (4.9%) were sickle homozygous (SS), seven had sickle β-thalassemia (S-β thal): [2 babies had S-β^0^ thal (CD 15 (G→A), CD 41/42 (-CTTT); 5 babies had S-β^+^ thal (IVS 1→5 (G→C)], one baby had sickle-delta-beta thalassemia (HbS -δβ thalassemia) and two babies had HbSD disease. In addition, five babies with HbE trait, four with β-thalassemia trait, three with alpha chain variants (Hb Koya Dora, Hb Fonatinebleau and HbO Indonesia) and one case of HbH disease were also identified. The cohort of 104 SS babies (Males-59, Females-45) was followed up clinically every month for a year and then every 3 months for 3–4 years for hematological and clinical evaluation. However, in case of any symptoms they came earlier. 92% of the SS babies belonged to non-tribal communities while 8% belonged to tribal communities.

75 out of the 104 SS newborns, six of the seven S-β thal babies and the two babies with Hb SD disease could be followed up clinically while 29 (28%) were lost to follow up.

### Age at presentation

Sixty two of the 75 SS babies had some clinical complications while 13 babies remained asymptomatic. Among them, 12 babies (19%) presented within 6 months of age, 17 (28%) between 6 and 12 months of age, 25 (40%) between 1 and 2 years of age while 8 babies presented after 24 months. The 2 Hb SD disease babies also presented within the first 6 months of life.

**Fig 1:**
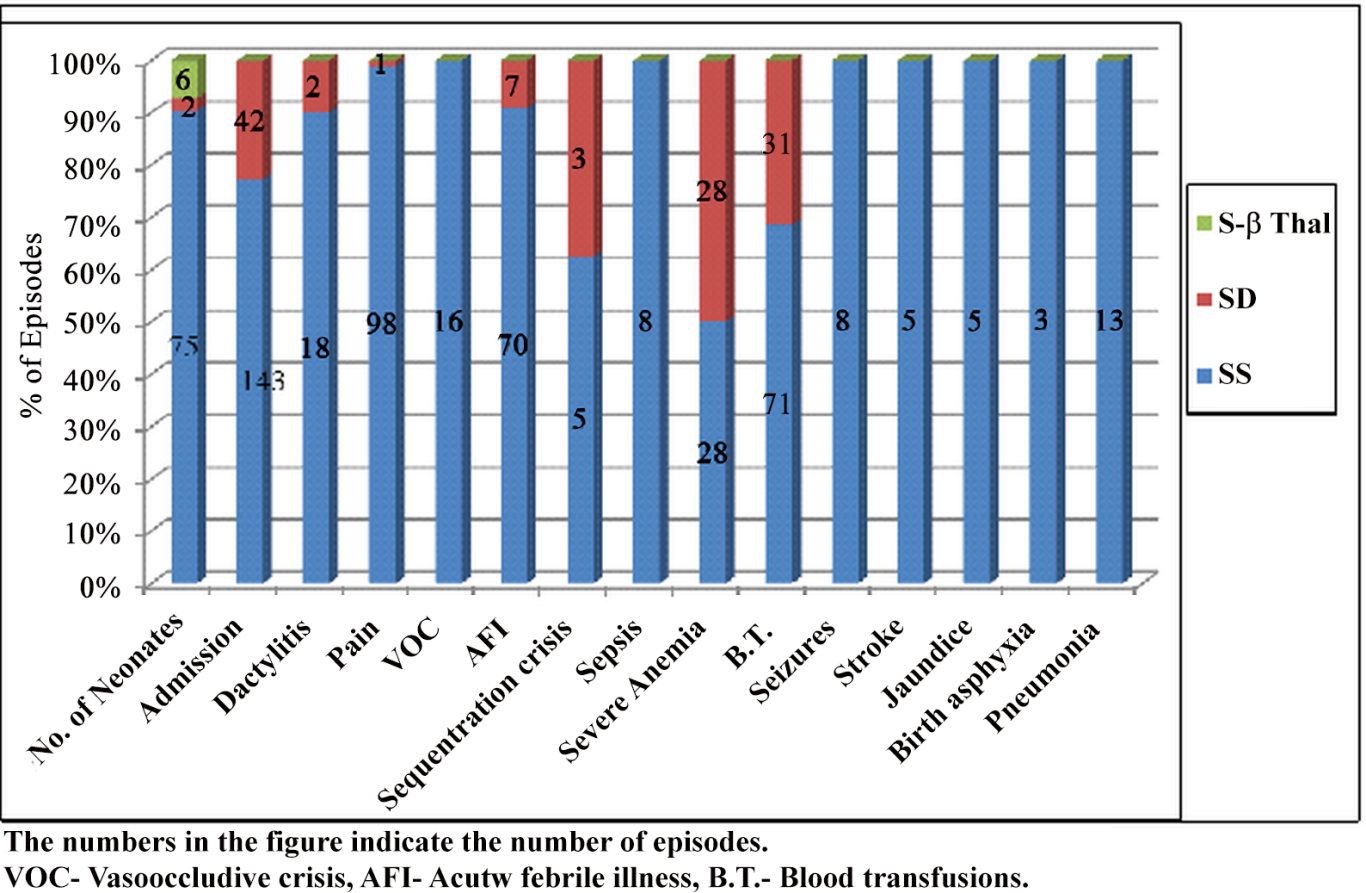
Clinical events observed in SS,S-βthalassemia and HbSD newborns on follow up. The numbers in the figure indicates the number of episodes. VOC-Vasoocclusive crisis, AFI- Acute febrile illness, B.T.- Blood transfusions.

The clinical events observed in the SS, S-β thal and SD babies are summarised in Fig 1.

### Acute sickle crisis

Table 1 illustrates the clinical events observed in this SS cohort. Painful events followed by blood transfusions and acute febrile illness were the main clinical complications with the overall incidence of 59.7, 45.1 and 42.6 per 100 person years. Vasoocclusive crisis (VOC) was observed at 9.7 per 100 person years, sequestration crisis was observed at 3.0 per 100 person years while death was observed at 3.65 per 100 person years. Dactylitis/Hand foot syndrome was seen in seven SS patients and one HbSD disease patient with a total of 20 episodes within 2 years of age and in the majority of the severe cases it was the 1^st^ clinical event observed. Acute splenic sequestration (ASS) crisis was seen in the first 2–3 years of life in two SS babies and one HbSD disease baby. One SS and one HbSD disease baby had three episodes each of sequestration crisis but splenectomy was not advised. Severe anemia requiring transfusions (3 or more/year) was the main clinical event in the two HbSD disease babies while the six sickle β-thalassemia babies had none of the above complaints. Acute chest syndrome (ACS) was not found in this cohort.

**Table 1:** Incidence of clinical events in SS cases per 100 person years.

Sr. No	Clinical Events	SS cohort per 100 person years (164 years of observation)
1.	Hospitalizations	87.2
2.	Painful events	59.7
3.	Blood transfusions	45.1
4.	Acute febrile illness	42.6
5.	Vasoocclusive crisis	9.7
6.	Sepsis	4.8
7.	Stroke	3.0
8.	Sequestration crisis	3.0
9.	Death	3.65

### Infections

Eight SS babies had sepsis with the overall incidence of 4.8 per 100 person years (Table 1) and two babies died due to sepsis. Microbial isolates were negative in these cases. Seven babies had sepsis in their initial days of life when Pen–V was not started and in one baby sepsis occurred at 15 months due to non-compliance. Eight SS babies also had pneumonia. Other infections included, right foot abscess with cellulitis and osteomyelitis. The microorganisms isolated in these cases included *Staphylococcus*, *E-coli and Klebsiella*.

### Siezures and Stroke

Seven SS babies had eight episodes of siezures and three babies had five episodes of stroke at 14, 42 and 30 months with an overall incidence of 3.0 per 100 person years (Table 1). One baby had repeatedly three episodes of stroke. The magnetic resonance imaging (MRI) scan was done in one patient and showed left cerebral and left temporal partial small acute infarct. All the three babies were put on chronic transfusion therapy to avoid recurrence of stroke, yet one baby had two more episodes of stroke. Transfusions were also given when the Hb levels dropped to less than 6.0g/dl.

### Hematological and hemoglobin analysis

The hematological and hemoglobin analysis at birth and at the last follow up (3–4 years) in SS babies is illustrated in Table 2. The mean Hb level at last follow up was 8.3 ± 1.0 g/dl and fetal hemoglobin (HbF) level was 21.4 ± 5.4% (Table 2).

**Table 2:** Hematological and hemoglobin analysis in SS cases. WBC-White blood cells, RBC-Red blood cells, Hb- Hemoglobin, MCV-Mean corpuscular volume, MCH- Mean corpuscular hemoglobin, MCHC-Mean corpuscular hemoglobin concentration, RDW-Red cell distribution width, HbA2- Hemoglobin A2, HbF- Fetal hemoglobin, HbS-Sickle Hemoglobin

	At Birth (1–7 days) (Mean ± SD)	Last follow up (3–4 years) (Mean ± SD)
WBC (x10^3^/μl)	9.6 ± 4.3	12.7 ± 5
RBC (x10^12^/l)	5.1 ± .5	3.2 ± 0.6
Hb (g/dl)	17.9 ± 2	8.3 ± 1.0
MCV (fl)	107. ± 8	85 ± 11.0
MCH (pg)	34.7 ± 3	26.5 ± 3.0
MCHC (g/dl)	33 ± 1.5	31.4 ± 2.1
RDW (%)	19.0 ± 2	23.5 ± 4.0
HbA_2_ (%)	0.21 ± 0.44	3.67 ± 2.05
HbF (%)	89.9 ± 9.4	21.4 ± 5.4
HbS (%)	14.8 ± 3.9	73.5 ± 6.4

### Haplotypes

Out of the 150 SS chromosomes, 141 (94.0%) were linked to the Arab-Indian haplotype, six were linked to the Bantu A2 and three to an Atypical1 haplotype (+ - + - + + + + -). Of the six babies having the Arab-Indian/Bantu A2 haplotype, two patients showed a moderately severe clinical course and one baby died due to severe anemia. Of the three babies with the Arab-Indian/Atypical1 haplotype, one could not be followed up and two patients showed a mildly severe clinical course.

### Effect of alpha thalassemia on the clinical presentation

Alpha genotyping was done in 69 of the 75 SS babies. 50 babies (72%) had a normal alpha genotype while 19 babies (28%) had alpha gene deletions (-α^3.7^/αα = 12, -α^4.2^/αα = 6, -α^3.7^/-α^4.2^ = 1). The incidence of clinical events per 100 person years was determined in babies with and without alpha thalassemia (Table 3). Infants with alpha thalassemia had a significantly higher incidence of painful events, sepsis and dactylitis. All the six sickle –β thalassemia babies had a normal alpha genotype (αα/αα) while the alpha genotypes in the two HbSD disease babies were αα/αα and -α^4.2^/αα.

**Table 3:** Comparison of clinical events in Hb SS patients (Cases per 100 Person years) with and without alpha thalassemia.

Clinical events	SS (n = 50) without α thalassemia 125 yrs of observation	SS (n = 19) with α thalassemia 47.5 yrs of observation	p-value
Painful events	51.2	71.6	p<0.0001
Vasoocclusive crisis	8	8.4	p = 0.08
Sepsis	2.4	10.5	p<0.0001
Dactylitis	8.8	14.7	p = 0.002
Severe Anemia	15.2	14.7	p = 0.02

### Effect of HbF on disease severity

HbF levels in the SS cohort varied from 3.3 to 37.2% (mean 21.4 ± 5.4%). Based on an earlier study, [14] a cut off of 20% of HbF was taken to determine the effect of HbF on the clinical parameters in the SS babies. HbF levels were estimated in 50 SS infants over 3–4 years of age and 29 of them (58%) had HbF levels greater than 20% while 21 (42%) had HbF levels less than 20%. A statistically significant inverse association of HbF was seen with VOC and severe anemia (p = 0.0002, p = 0.009). Association of HbF with dactylitis, painful events and hospitalizations was not statistically significant. The HbF levels in two SS and one HbSD disease patients who had sequestration crisis were 22.8, 16.0 and 6.3% respectively while the HbF levels in three SS patients with stroke were 17.0, 11.0 and 3.3%.

### Mortality

Six SS babies (8%) died during the follow up with the overall mortality rate of 3.65 per 100 person years (Table 1). In three cases (ages- 14 months, 24 months and 2 days) the cause of death was unclear. One baby died at 24 months due to hypoxic encephalopathy, 2 babies died of sepsis (20 days and 3 months). Five of the six SS babies died at home or on their way to the hospital.

## Discussion

Neonatal screening helps to recognize the early signs of the disease and minimize morbidity and mortality by early comprehensive care and prophylactic treatment. Our targeted newborn screening approach in central India identified 4.9% of SS babies along with other hemoglobinopathies. The above babies were enrolled at the sickle cell clinic and were given early comprehensive care and prophylactic treatment. Few babies with HbS-β thalassemia may have been missed as universal screening was not done.

Vichinsky et al demonstrated that the overall mortality rate for American patients with sickle cell anemia diagnosed in the neonatal period was 1.8% compared with 8% for children diagnosed after three months of age [15]. In the Jamaican Sickle Cell Cohort Study, 14% of children died in the first two years of life when early age interventions were not implemented compared to less than 1% when the preventive strategies were available [16]. Neonatal hemoglobinopathy screening of 191 783 newborns in Belgium identified 123 babies with SCD of whom 1 died of septicaemia when the prophylactic treatment was interrupted [17].

Such established newborn screening programs have not been initiated in India although the load of SCD is very high in central India. An initial pilot effort was made in Raipur district in Chhattisgarh in India, on screening few babies (1,158), however the affected babies were not followed up [18]. Screening of sickle cell disease in newborns was also undertaken in Kalahandi district in Odisha in eastern India which has a large tribal population. Thirteen of the 761 newborns screened were homozygous for sickle cell disease. This study showed the feasibility of undertaking newborn screening in a district hospital which helped to identify a hot spot area for sickle cell disease in this region [19].

Another recent study on newborn screening among tribal populations in South Gujrat in Western India showed that 21.8% of SCD babies had severe clinical complications before 5 years of age [20]. Our previous study was the first newborn screening (NBS) program in central India for early diagnosis and timely prophylactic treatment [5]. In that study we tried to look at prevalence of sickle cell anemia and trait among the newborns from central India and we identified sickle cell anemia in 1.1% neonates with the highest incidence in the scheduled caste group. This was the first large study reporting the incidence of sickle cell anemia by newborn screening from India. In the present study, we raised a cohort of sickle cell disease babies identified by newborn screening and followed them up regularly to record the early clinical events and the hematological presentation and tried to look for possible correlation with different genetic modifiers. Thus we have evaluated the morbidity and mortality of these babies in the first 3 to 5 years of life. The overall mortality in this study with the SS babies was 3.65 per 100 person years while the mortality rate due to complications arising from sickle cell disease was 1.8 per 100 person years. All the deaths occurred before 2 years of age and 3 of the SCD related deaths were due to sepsis and hypoxic encephalopathy (in 3 SS babies the cause of death was unknown). The mortality rate in our cohort was higher as compared to the mortality rate in the Cooperative Study of Sickle Cell Disease (CSSCD) cohort which was 1.1 per 100 person years and the Dallas cohort which was 0.6 per 100 person years [13, 21]. The reason for the higher mortality rate in our study may have been due to inaccessibility to immediate care as these families belonged to an economically disadvantaged society and were residing in remote areas. This could also be one of the reasons why 28% of our patients were lost to follow up. Moreover, our preliminary experience is on fewer infants with a shorter follow up period than the earlier studies.

Since all painful events do not lead to VOC, pain and VOC were considered as separate complications. Pain followed by acute febrile illness and severe anemia with blood transfusion requirements were the major complications observed in this study (Table 1). Painful crisis was also the most common sickle related event in SS patients of the Cooperative Study [13]. Many studies have been undertaken to evaluate the influence of HbF levels on painful crisis which have shown conflicting results. Baily et al showed that abdominal painful crisis was not related to HbF concentration [22] whereas Baum et al showed a gender bias with painful crisis affecting mainly male patients [23]. Platt et al demonstrated that even a small increase in HbF levels could reduce pain and improve survival [11].The study of Donaldson et al observed no association between low Hb F levels and increase in clinical severity of the disease and concluded that low HbF levels may be protective against leg ulcerations[24]. Similar results were reported by Powars et al 1980 [14]. In the present study also we did not find any significant effect of HbF on painful events but a statistically significant association was observed with VOC.

As noted by Miller et al “Three easily identifiable manifestations of sickle cell disease that may appear in the first two years of life (dactylitis, severe anemia, and leukocytosis) can help to predict the possibility of severe sickle cell disease later in life”[25]. Severe anemia (Hb < 5g/dl) requiring transfusions was seen in 24% of the patients in our study with the hemoglobin levels dropping to 3–5 g/dl. A statistically significant inverse effect of HbF was seen in patients with severe anemia. Since dactylitis was the first complication seen in majority of our severe patients it was considered as an independent complication. It has been reported in 50% of children by the age of 2 years in the Jamaican Cohort Study [26]. The cohort study from Guadeloupe also indicated that 13 of the 14 children who had dactylitis before 6 months of age had at least one severe event later in life indicating dactylitis as a predictive factor for an adverse outcome [27]. 9% of our SS patients and 1 HbSD disease patient had 20 separate episodes of dactylitis, the time interval between each varying from two to seven months.

Acute chest syndrome and pneumonia are very difficult to differentiate in babies and pneumonia being one of the precipitating factors for ACS, this complication in the babies was labelled as pneumonia. ACS followed by ASS and sepsis were the major cause of mortality in Jamaica [28]. ACS was not found in the babies in our cohort but the incidence of sequestration crisis was 3.0 per 100 person years. It has been shown that ASS may commence as early as 3 months of age but is most common in the second 6 months of life; two-thirds of attacks occurring before 2 years of age and the events become rare after 6 years of age with the recurrence rate being 47% [29]. In our cohort, ASS crisis occurred between 2–3 years of age while one SS and one HbSD case had three episodes each of sequestration crisis.

Infection particularly pneumococcal sepsis is the leading cause of death in children with SCD [12, 30]. Sepsis was diagnosed on the basis of fever, inappropriate tachycardia, increased respiratory rate, toxic granulation with neutrophil leucocytosis, Dohle bodies in the blood smear and increased CRP levels. Eight babies had sepsis in our cohort and two of them died. In these cases either Pen V was not started or they were not compliant to the therapy. Eight SS children also had pneumonia, while the organisms isolated in other infections included *Staphylococcus*, *E-coli and Klebsiella*. This is in contrast to the CSSCD cohort where infections occurred most commonly due to *Streptococcus pneumoniae* and *Hemophilus influenzae* and caused 11 deaths [13].

Stroke is a feature of early childhood with an incidence of 8% by 14 years of age in the Jamaican cohort [2]. Three of our children (4%) under three years of age had five episodes of stroke with one child having three episodes. A recurrence rate of 67% had been observed in patients with cerebral infarction within 36 months of the initial event [31–33]. The mean HbF level in these patients was 10.4% suggesting that lower levels of HbF might have increased the chance of stroke in these children.

SCD patients with alpha thalassemia had a higher risk of painful events, sepsis and dactylitis and lower incidence of severe anemia (Table 3). Our findings are comparable with the CSSCD cohort study [13]. Platt et al demonstrated no effect of α thalassemia on the pain phenotype [11].

The hematological profile in the SS patients showed that anemia was apparent from 6 months of age. An increase in the HbA_2_ levels at the last follow up was observed due to the presence of HbS adducts which tend to increase the actual HbA_2_ levels on HPLC. The HbF levels at 3–4 years were 21.4 ± 5.4%, majority of the sickle chromosomes being linked to the Arab- Indian haplotype.

## Conclusion

In conclusion, this is the first newborn SCD cohort in central India to be systematically evaluated for 3–4 years. It emphasises the importance of early diagnosis and prophylactic treatment, parental education and the importance of tertiary care centres that should be made available to allow for prompt treatment. We have also shown recently that 75.4% adult SCD patients in India with the Arab Indian haplotype also have severe manifestations [34]. Thus SCD in India presents with very variable phenotypes. This study is also the first attempt to understand the natural history of SCD in central India. It highlights the fact that SCD in Indian patients although linked to the Arab Indian haplotype can have an early presentation and severe manifestations and comprehensive care could reduce morbidity and mortality.
